# Research on Recognition of Road Hypnosis in the Typical Monotonous Scene

**DOI:** 10.3390/s23031701

**Published:** 2023-02-03

**Authors:** Huili Shi, Longfei Chen, Xiaoyuan Wang, Bin Wang, Gang Wang, Fusheng Zhong

**Affiliations:** 1 College of Electromechanical Engineering, Qingdao University of Science & Technology, Qingdao 266000, China; 2 Collaborative Innovation Center for Intelligent Green Manufacturing Technology and Equipment of Shandong, Qingdao 266000, China

**Keywords:** road hypnosis, monotonicity effect, active safety warning, machine learning

## Abstract

Road traffic safety can be influenced by road hypnosis. Accurate detection of the driver’s road hypnosis is a very important function urgently required in the driver assistance system. Road hypnosis recurs frequently in a certain period, and it tends to occur in a typical monotonous scene such as a tunnel or a highway. Taking the scene of a tunnel or a highway as a typical example, road hypnosis was studied through simulated driving experiments and vehicle driving experiments. A road hypnosis recognition model based on principal component analysis (PCA) and a long short-term memory network (LSTM) was proposed, where PCA was used to extract various parameters collected by the eye tracker, and the LSTM model was constructed to identify road hypnosis. The accuracy rates of 93.27% and 97.01% in simulated driving experiments and vehicle driving experiments were obtained. The proposed method was compared with k-nearest neighbor (KNN) and random forest (RF). The results showed that the proposed PCA-LSTM model had better performance. This paper provides a novel and convenient method to realize the driver’s road hypnosis detection function of the intelligent driver assistance system in practical applications.

## 1. Introduction

Road traffic safety is an important issue of widespread concern in modern society. Human attributes are the main causes of traffic accidents around the world [1]. According to an accident causal analysis of the Natural Driving Dataset funded by the National Academy of Sciences [2], nearly 90% of accidents are driver-related. To reduce the occurrence of traffic accidents, smart car manufacturers in various countries are trying to take a variety of measures, which include both active and passive safety measures. As an active safety subsystem, the advanced driving assistance system is considered to be an effective means to avoid traffic accidents to the greatest extent. On the one hand, the environment can be monitored in real time with various sensors installed outside the vehicle. The influence of other vehicles nearby can be mastered by perceiving the multi-source information of surrounding vehicles in time. On the other hand, the various states of the driver can be monitored with sensors inside the vehicle. The abnormal driving behavior of the driver can be identified, and corresponding measures can be taken to prevent accidents. The driver state performance is influenced by different human attributes, including driving propensity [3,4], driving emotion [5,6], driving intention [7], behavioral patterns [8], and so on. The research on active safety has received the most attention in advanced driver assistance systems. One of the core concepts is driving behavior identification. Accurate identification of various driving behaviors is one of the most effective technical means to prevent traffic accidents, which plays an important role in improving road traffic safety [9]. In the research and application fields of abnormal driving behavior identification, such as fatigue driving [10,11], distracted driving [12,13], drunk driving [14], and emotional driving [15], a great deal of research results have been achieved, some of which have been applied to the current advanced driver assistance system. Deeply understanding various types of characteristic information of the driver and accurately identifying the specific mental state of the driver is one of the most serious challenges in the field of intelligent transportation, which will be the case for a long time to come.

In recent years, studies of many researchers in the field of intelligent transportation have been shifted from traditional road simulation and vehicle data to human factors in real vehicle driving tasks. The research on driving behavior involves multi-disciplinary fields such as ergonomics, cognitive neuroscience, and machine vision. There have been many studies on the classification and monitoring of the driver’s mental state. According to the research of the American Automobile Association Foundation for Traffic Safety (AAA FTS), the driver’s state of consciousness is divided into the following five categories: attention, distraction, looked but not seen, drowsy, and unknown. Many studies have paid attention to the fatigue and distraction states of drivers, and only a few studies have involved road hypnosis, a special state that can have an important influence on road traffic safety. Road hypnosis is a state that is extremely likely to appear in driving tasks, especially in monotonous scenes or roads with frequent repetitive and familiar scenes (such as scenes of tunnels or highways). The specific manifestations of road hypnosis are transient amnesia, trance, hallucinations, decreased vigilance, inattention, and being in a state of unconscious driving. In this case, although the driver can maintain a normal driving state, the reaction speed will be significantly slower than that under the normal driving state. Especially in tunnels and highways, which are prone to road hypnosis, cars often travel at high speeds. Once an accident occurs, it is often more serious than a conventional road traffic accident. Therefore, it is necessary to conduct in-depth research on road hypnosis.

As a state that tends to appear in a monotonous or familiar environment and is prone to occur, strengthen, maintain, transfer, and disappear frequently within a period, road hypnosis is essentially different from driving fatigue and driving distraction. Driver fatigue is defined as a transitional state from wakefulness to sleep, in which the driver’s alertness, mental function, and physical and mental function decrease due to external environmental factors and physiological factors. Meanwhile, sleep-related sensory or physical symptoms, such as frequent eye blinking, yawning, and abnormal head posture, may occur. Driver distraction is a state in which the driver shifts his or her attention from the main task of driving to secondary tasks unrelated to driving, which usually includes visual distraction, cognitive distraction, auditory distraction, and motor distraction. Both aforementioned abnormal driving behaviors have similarities with the road hypnosis state, but they are different in their internal mechanism and external performance. Compared with driving fatigue, road hypnosis is less severe, and it occurs repeatedly within a period. When a driver is in a state of road hypnosis, he often does not perceive that he is in a state of road hypnosis subjectively, which is in an unconscious driving state. When a driver emerges from road hypnosis, there is usually a noticeable state of alertness. Although the driver may not remember what happened to him under road hypnosis, he has a clear memory of having recently experienced the above-mentioned stupor state. Driving fatigue usually makes drivers feel tired subjectively. Once it appears, it will be in a persistent state, and the driver needs to take appropriate measures to overcome it before it subsides. Compared with driving distraction, road hypnosis is similar to cognitive distraction, in which the driver’s attention is not focused on the main task of driving. However, the difference is that when the driver is in road hypnosis, he or she is still performing the main task of driving. However, in the state of cognitive distraction, the driver is performing corresponding subtasks that have nothing to do with driving. The state of road hypnosis is, to a greater extent, affected by the main driving task itself and the road environment (monotonous, familiar, and repetitive), so it is a sudden phenomenon that occurs frequently within a period. However, the current research has not been able to accurately and effectively identify the hypnotic state by the road and lacks a valuable theoretical framework and applicable technical solutions from the perspective of the human–vehicle–environment system.

As typical monotonous scenes, tunnels and highways are the two road environments more prone to lead to road hypnosis. In this paper, a typical monotonous scene was selected. Taking tunnels and highways as examples, the road hypnotic state of drivers in simulated driving and vehicle driving was studied. PCA [16] and LSTM [17] were used to establish the identification model of road hypnosis to identify the corresponding states of drivers in simulated driving and vehicle driving experiments. The accuracy rate was 97.01% according to the dataset obtained from the simulated driving experiment, and a 93.27% accuracy rate was achieved according to the dataset obtained from the vehicle driving experiment.

## 2. Literature Review

There are few studies on aspects of its generation, enhancement, maintenance, transfer, and disappearance. Williams G.W. et al. [18] descriptively defined road hypnosis in 1963. They described it as an amnesiac, trancelike state with a longer reaction time, in which, however, a normal effect of driving was performed. Meanwhile, they noted the difference between hypnosis and sleep. Shor R.E. et al. [19] set 10 triggers in a monotonous highway scene, and asked drivers to perform continuous and repetitive tracking or monitoring tasks. They demonstrated the existence of road hypnosis on the highway by designing a reaction time detection device and described it as a state of fantasy, distorted thinking, and mental fatigue. Williams G.W. et al. [20] went a step further in 1970 to induce road hypnosis by gazing at bright spots on a monotonous highway and described it as a state of drowsiness and distorted thinking and judgment. Wertheim A.H. et al. [21] proposed the eye movement theory. They emphasized that it was not only the monotonous environment that triggered road hypnosis, but also the predictability of the environment. Cerezuela G.P. et al. [22] induced road hypnosis by asking drivers to drive in a highly predictable environment for a long time. They described road hypnosis as an involuntary driving pattern and used relative parameters in statistics to identify road hypnosis.

LSTM is a special form of recurrent neural network, which is proposed to solve the problem of gradient disappearance and gradient explosion during long sequence training [17]. In 1999, Felix A. Gers et al. [23] introduced the forget gate mechanism in LSTM to avoid the network crash problem caused by not resetting the internal state of the network. In 2000, they further added the Peephone to the LSTM internal state unit to enhance the network’s ability to distinguish subtle features in the input sequence [24]. Alex Graves et al. [25] proposed a bidirectional long short-term memory neural network, BiLSTM, also known as vanilla LSTM, which is currently the most widely used LSTM model. LSTM is characterized by a time loop structure, which can well describe sequence data with temporal and spatial correlations, and is widely used in natural language processing and other fields. Google uses the model for speech recognition and for improving the machine translation capabilities of Google Translate. Amazon uses LSTM models to boost Alexa’s capabilities. Facebook has implemented the translation function based on the LSTM model for a long time. LSTM is also widely used in the research of driving behavior, including detecting driver fatigue, distraction, intention, and emotion [26]. Alameen Sara A. et al. [27] proposed a spatiotemporal model for monitoring drowsiness visual indicators from videos. The model depended on integrating a 3D convolutional neural network (3D-CNN) and LSTM. The model achieved test detection accuracy of 96%, 93%, and 90% on YawDD, Side-3MDAD, and Front-3MDAD. Cura Aslihan et al. [28] developed a LSTM model and a CNN model to classify and assess bus driver behavior characterized by deceleration, engine speed pedaling, and corner turn and lane change attempts. Wang Shu et al. [29] proposed a method based on LSTM for identifying the driver braking intention. Wollmer Martin et al. [30] presented a technique for online driver distraction, modeling the long-range temporal context of driving and head tracking data. LSTM networks enabled the reliable subject-independent detection of inattention with accuracy of up to 96.6%. Wu Zhanqian et al. [31] proposed an Attention-Enhanced Residual-MBi-LSTM (attention-enhanced bidirectional multi-layer residual long short-term memory neural network) model for lane change intention recognition based on the trajectory characteristics and vehicle interaction information, which can identify driver intention on average 2.07 s in advance.

## 3. Materials and Methods

### 3.1. Participants

Fifty drivers were recruited from society to participate in driving experiments. Among them, the ratio of male to female was 8:2, the age distribution was between 30 and 60 years old, and the driving age was distributed between 10 and 32 years. The basic information about the drivers in the experimental sample can be seen in Table 1.

### 3.2. Equipment

**Figure 1:**
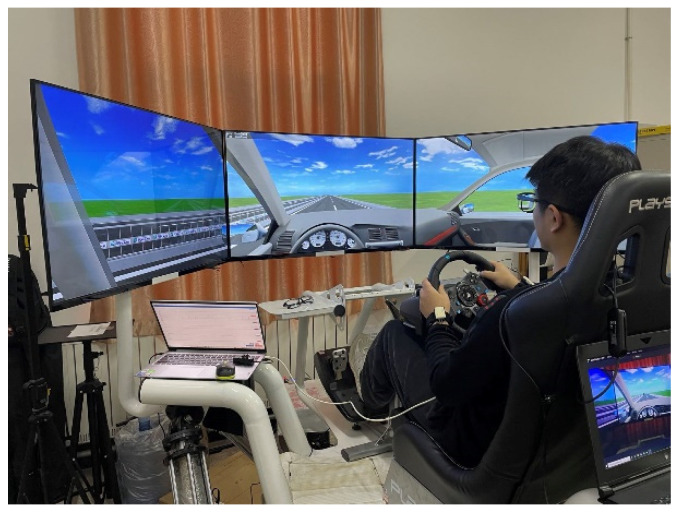
Experiments of simulated driving.

This study included simulated driving experiments and vehicle driving experiments. The experimental equipment used in the simulated driving experiment included a driving simulation system, eye tracker, human factor equipment, laptop computer, video recorder, etc. The experimental environment is shown in Figure 1. The simulated driving experiment was carried out in a laboratory environment with standard lighting and curtains. In the experiment, the eye tracker was used to collect the driver’s eye movement information, and the video recorder was used to record the whole process of the experiment. In the driving simulation system, the Logitech G29 driving kit and a six-degrees-of-freedom platform were used to simulate the driving of the vehicle. UC-win/Road software was used to generate a simulated driving environment. The simulated driving environment was displayed through three 55-inch triple display screens. Another three experimental assistants participated in the simulated driving experiment.

**Figure 2:**
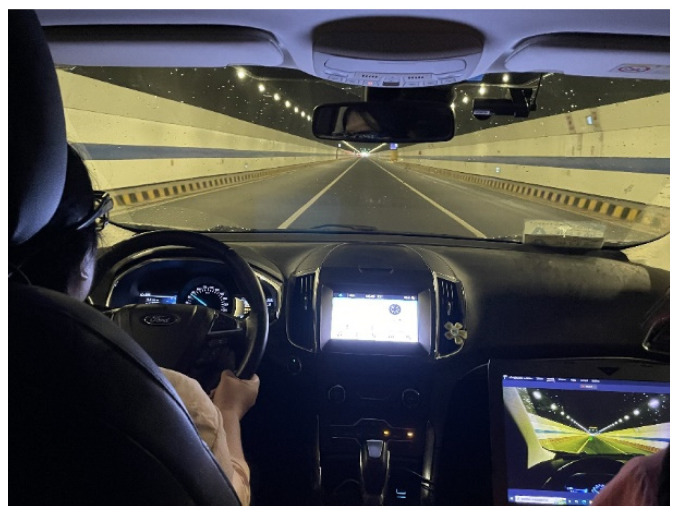
Experiments of actual driving.

The experimental equipment used in the vehicle driving test included a Buick car, eye tracker, human factor equipment, laptop computer, video recorder, etc. The experimental environment is shown in Figure 2. In the vehicle driving experiment, the eye tracker was used to collect the driver’s eye movement information. A video recorder was used to record the whole experiment process. Another three experimental assistants participated in the vehicle driving experiment.

A relatively comprehensive experiment was conducted, intending to collect various characteristics of drivers under road hypnosis. Many types of human factor equipment were used in the experiment, including ECG equipment, myoelectric equipment, respiratory rate equipment, heart rate equipment, skin temperature equipment, and blood oxygen equipment. However, in this study, we used ECG parameters and eye movement parameters, combined with the driver’s external performance, to determine whether the driver was in the road hypnosis state. Whether the result was correct or not could be judged through the active inquiry of the experimental auxiliary staff. Other human factor parameters were not used in this study.

### 3.3. Procedure

In the monotonous driving environment, the change in road alignment is simple, the traffic flow is small, and the road environment lacks changes, and there is little stimulation of the visual senses. This leads to driver distraction and a reduction in operating ability and driving alertness, which is likely to activate road hypnosis after driving for a while. Therefore, the highway scenario was selected to carry out the simulated driving experiment in this study. In the vehicle driving experiment, due to the fast speed of the vehicle on the highway, it is especially easy to encounter other fast-speed interfering vehicles. The safety of vehicle driving experiments cannot be well guaranteed. Therefore, a tunnel scene with fewer vehicles and slower vehicle speeds than that of the expressway was selected for the vehicle driving experiment.

The processes of the simulation driving experiment and vehicle driving experiment were basically the same, as follows:

- Select the road environment for the experiment;
- Select the participants and determine the experiment time;
- Before the experiment, the experimental auxiliary staff should debug the experimental equipment;
- Start the driving experiment, and assist the experiment personnel to complete various tasks in the experiment and collect relevant data;
- Finish the experiment and arrange the equipment by the auxiliary staff.

#### 3.3.1. Simulated Driving Experiment

To ensure that the driving environment generated by the simulation software was similar to that of the actual highway, a two-way four-lane highway scenario was selected for the simulated driving experiment in this paper. The whole road section was composed of straight road sections, with a total length of 40 km and a single lane width of 3.75 m. The scene that we designed in the simulated driving experiment was similar to the highway scene on the actual road. The road surface was smooth and open, without interference from pedestrians and other vehicles. There was no water, gravel, or other obstacles on the road surface, and there was no interference from buildings, bushes, pedestrians, or others on both sides of the road. Trees and grass on both sides of the road were added at regular intervals. This segment was repeated throughout the experiment until the end.

It was required that the subjects experienced no sleep disturbance, and, meanwhile, they were to ensure sufficient and regular sleep in the three days before the experiment. The degree of myopia of the experimenters did not exceed 600 degrees. The simulated driving test time was 9:00–11:00 in the morning. During the experiment, three experimental assistants were required. Before the simulated driving test began, the test assistants debugged the equipment. During the experiment, the subjects were required to wear eye trackers and drive in a single lane at a speed of 120 km/h for 20 min. Any problems during this process could be reported to the experimental assistants. A special experimental assistant was in charge of recording and observing the changes in the driver’s ECG in real time. They observed the driver’s external performance state when the driver’s ECG characteristics were stable. When the driver appeared in a state in which they might wake up from road hypnosis, the experimental assistant asked the driver whether the state occurred and recorded it. After the driving process was over, the subjects took off the eye tracker and rested for ten minutes, and the experimental assistants checked and debugged the equipment. During this period, an experimental assistant asked the subjects whether they had fatigue, distraction, and other behaviors during the driving process they had experienced and recorded them. Combining the whole video and the eye movement information video of the driver during the experiment, at the time point when road hypnosis may have appeared during the driving process, the driver was asked whether the road hypnosis occurred and it was recorded. After the break, the subjects wore eye trackers and kept driving in a single lane at a speed of 120 km/h. The driving time was 40 min under the condition without talking to other people. This process was consistent with the experimental process performed previously. After the simulated driving was completed, the experimental assistants organized the equipment and ended the experiment.

#### 3.3.2. Vehicle Driving Experiment

In the vehicle driving experiment, the road section of the Qingdao Binhai Highway (Qingdao University of Science and Technology Laoshan Campus—Shandong University Qingdao Campus) was selected for the experiment. The total length of the experimental road section was 36 km, and the road speed limit was 80 km/h. The environment along the road was relatively monotonous. The Yangkou Tunnel, passed by on the route, had a total length of 7.76 km, with a two-way six-lane arrangement, and was divided into left and right tunnels, among which the left line was 3.875 km long, and the right line was 3.888 km long. The single hole was 14.8 m wide and 8 m high.

In the vehicle driving experiment, considering the impact of the morning rush hour, when there were too many road vehicles, the period from 10:00 to 12:00 was selected for the experiment. Excluding the subjects, a total of three experimental assistants participated in the vehicle driving experiment. The road conditions in the first 5 km of the actual vehicle test road section (Qingdao University of Science and Technology Laoshan Campus-Ocean University of China Laoshan Campus section) were not particularly monotonous. During the driving process of this road section, the test personnel were familiar with the vehicle driving environment. Subjects could report or ask any questions to the experimental assistants. After arriving at the Laoshan Campus of Ocean University of China, participants stopped for a 5-min rest, and then continued the driving process. The driver drove at a constant speed while ensuring driving safety. Drivers were required to avoid overtaking and changing lanes as much as possible during driving and could not to talk to other people. During this process, the experimental assistants collected the eye movement information of the subjects through the eye tracker. At the same time, another experimental assistant videotaped the whole process of the subjects and observed the changes in the driver’s ECG in real time. They observed the driver’s external performance state when the driver’s ECG characteristics were stable. When a state of waking up from road hypnosis might have appeared, the experimental assistant asked the driver whether the state occurred and recorded it. After arriving at the Qingdao Campus of Shandong University, the subjects took off the eye tracker and rested for 20 min. An experimental assistant immediately asked the subjects whether they had experienced fatigue, distraction, and other behaviors during the driving process and recorded them. Combining the whole video and the eye movement information video of the driver during the experiment, at the time point at which the road hypnosis may have appeared during the driving process, the driver was asked whether the road hypnosis had occurred and this was recorded. During this period, the experimental assistants saved the data and debugged the equipment. After the break, the subjects put on the eye tracker again and returned from the Qingdao Campus of Shandong University to the Laoshan Campus of Qingdao University of Science and Technology. The flow of this process was consistent with that from the Laoshan Campus of Ocean University of China to the Qingdao Campus of Shandong University. After arriving at the destination, the experimental assistants sorted out the equipment and ended the experiment.

### 3.4. Data Collection and Analysis

In this paper, the glasses-type eye-tracking equipment developed by the 7invensun Company was used to collect eye movement information about the subjects. The collected parameters included basic information about the subjects, pupil information, gaze point information, eye saccade information, blink information, etc. The specific parameters and their meanings are shown in Table 2.

### 3.5. Model

**Figure 3:**
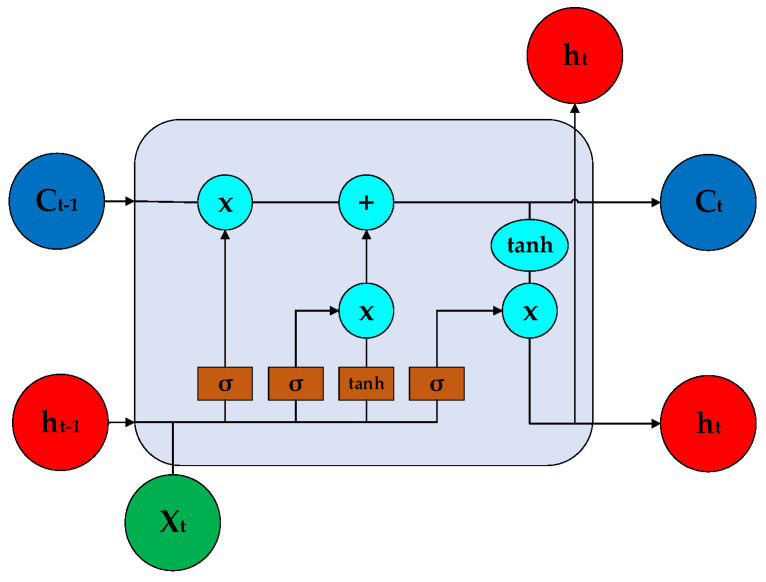
The structure of LSTM.

Based on the fact that the experimental data collected in this study were the data information with time series characteristics, the long short-term memory (LSTM) algorithm was used to establish a road hypnosis identification model. The structure of LSTM is shown in Figure 3.

LSTM mainly includes three gates, which are the input gate, forget gate, and output gate. 

**Input gate:** The input gate is used to update the cell state. First, the information of the hidden state of the previous layer and the information of the current input are passed to the sigmoid function. We adjust the value within 0~1 to decide which information to update. Here, 0 means not important, and 1 means important. Secondly, the information of the hidden state of the previous layer and the information of the current input must be passed to the tanh function to create a new vector of candidate values. Finally, the output value of the sigmoid function is multiplied by the output value of the tanh function, and the output value of sigmoid will determine which information in the output value of the tanh function is important and needs to be preserved.

**Forget gate:** This determines which information should be discarded or kept. The information from the previous hidden state and the current input information are passed to the sigmoid function at the same time, and the output value is between 0 and 1. A closer value to 0 means that it should be discarded, and a value closer to 1 means that it should be retained.

**Output gate:** The output gate is used to determine the value of the next hidden state, which contains the information of the previous input. First, we pass the previous hidden state and the current input into the sigmoid function, and then pass the newly obtained cell state to the tanh function. Finally, the output of the tanh function is multiplied by the output of the sigmoid function to determine which information the hidden state should carry. Then, we use the hidden state as the output of the current cell, and pass the new cell state and new hidden state to the next time step.

The calculation formulas of the forget gate, input gate, and output gate are as follows:

(1) $$f_{t}=\sigma (W_{f}\cdot [h_{t-1},x_{t}]+b_{f})$$

$f_{t}$ is the forget gate. σ is the gate activation function. $h_{t-1}$ is the output at the previous time step (*t* − 1). $x_{t}$ is the current input. $W_{f}$ is the weight associated with $h_{t-1}$ and $x_{t}$, while $b_{f}$ is the bias term.

(2) $$\begin{array}{l} {\tilde{C}}_{t}=\tanh(W_{C}\cdot [h_{t-1},x_{t}]+b_{C})\\ i_{t}=\sigma (W_{i}\cdot [h_{t-1},x_{t}]+b_{i}) \end{array}$$

$W_{C}$ and $W_{i}$ are the weights associated with $h_{t-1}$ and $x_{t}$. $h_{t-1}$ is the output at the previous time step (*t* − 1). $x_{t}$ is the current input. σ is the gate activation function. ${\tilde{C}}_{t}$ is the block input. $i_{t}$ is the input gate. $b_{C}$ and $b_{i}$ stand for the bias weight vector.

(3) $$C_{t}=f_{t}C_{t-1}+i_{t}{\tilde{C}}_{t}$$

$C_{t}$ is the output gate. $f_{t}$ is the forget gate. $i_{t}$ is the input gate. ${\tilde{C}}_{t}$ is the block input, while $C_{t-1}$ is the block input at the previous time step (*t* − 1).

In addition, the problems regarding gradient disappearance and gradient explosion have also been properly solved in LSTM. Its calculation formula is as follows:

(4) $$\begin{array}{l} \frac{\partial C^{\left(k\right)}}{\partial C^{\left(k-1\right)}}=C^{\left(k-1\right)}\sigma^{\prime }(\cdot )W_{f}o^{\left(k-1\right)}\tanh^{\prime }(C^{\left(k-1\right)})+a^{\left(k\right)}\sigma^{\prime }(\cdot )W_{i}o^{\left(k-1\right)}\tanh^{\prime }(C^{\left(k-1\right)})\\ +\;i^{\left(k\right)}\tanh^{\prime }(\cdot )W_{c}o^{\left(k-1\right)}\tanh^{\prime }(C^{\left(k-1\right)})+f^{\left(t\right)}\\ \prod_{k=t+1}^{T}\frac{\partial C^{\left(k\right)}}{\partial C^{\left(k-1\right)}}=(f^{\left(k\right)}f^{\left(k+1\right)}\cdots f^{\left(T\right)})+other \end{array}$$

## 4. Results

### 4.1. Principal Component Analysis Process and Results

The data obtained from the experiment were sorted out, the effectiveness of the driver’s road hypnotic state excitation was judged by the expert scoring method, and 50 sets of vehicle driving test data and 50 sets of simulated driving test data were obtained. The expert scoring method, also known as the analytic hierarchy process, is a qualitative and quantitative method for calculating weights. It uses the method of pairwise comparison to establish a matrix, and uses the relativity of the number size, with the principle that the larger the number is, the more important it is, and the higher the weight will be, and finally the importance of each factor is obtained. This is mainly completed by experts (here, we selected personnel who had researched in this area in our laboratory, including professors, scientific research assistants, Doctoral and Master’s students, etc., with a total of 8 personnel) in road hypnosis according to the video playback and experimental data of the experiment. The effectiveness of state excitation is scored. The basis for scoring includes a variety of influencing factors, such as whether the driver’s state in the video is fatigued, the driver’s reaction time after receiving the question, changes in the driver’s eye movement and ECG characteristics, etc. Using this method can eliminate the experimental data of insufficient stimulation of the road hypnosis state.

The experts screened 10 min of time segments with typical road hypnosis phenomena from the collected data as the road hypnosis driving state dataset. Because the state of road hypnosis is a state that reappears many times within a certain period, it is not a state that can last for a long time after it appears. Therefore, the data in the 10 min screened are not a completely continuous period. Ten minutes of normal driving data in a non-hypnotic state are selected as the normal driving dataset.

After the dataset screening is completed, another expert will manually score the selected dataset. They judge the validity of the dataset based on the video playback and confirm its final score. Due to the physical strength of the test subjects and the irregular driving posture in some experimental data, some drivers experienced driving fatigue. Therefore, the experimental data of nine drivers were excluded from the vehicle driving experimental data. Finally, 35 sets of effective video data were screened out to build a road hypnosis vehicle driving experiment database. They contained 25 sets of normal driving test data and 10 sets of road hypnosis driving test data. In the simulated driving experiment, the experimental data of three drivers were eliminated, and 43 sets of effective video data were screened out to construct the road hypnosis simulated driving experiment data set. They contained 27 sets of normal driving test data and 16 sets of road hypnosis driving test data.

**Figure 4:**
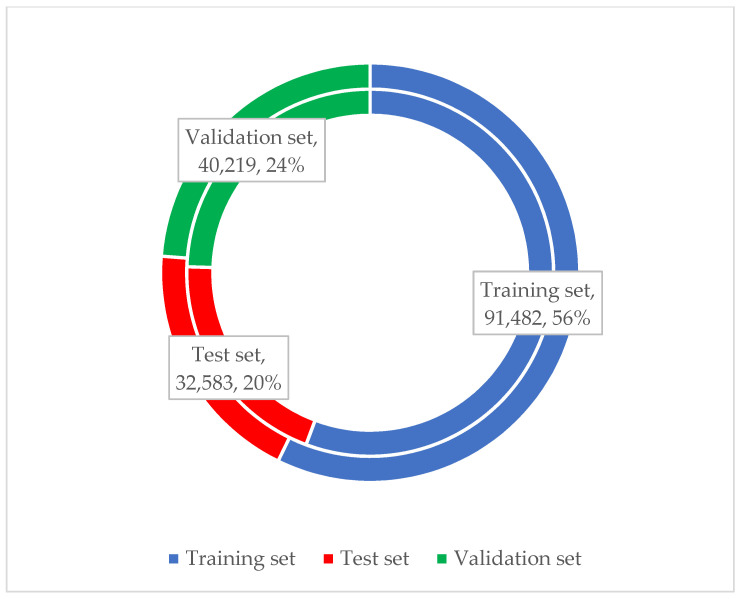
Ratio of training set, test set, and validation set.

The experimental data were preprocessed according to the above method, and a total of 183,517 simulated driving test data and 139,458 vehicle driving test data were obtained. They contained 164,284 valid simulated driving test data and 112,494 valid vehicle driving test data, respectively. Among them, 91,482 pieces of data were selected from the simulated driving test dataset to form the original database for model calibration and training. Meanwhile, 32,583 pieces of data were used for model testing. The remaining 40,219 pieces of data are used for model validation. In addition, 64,395 pieces of data were selected from the vehicle driving test dataset to form the original database for model calibration and training; 21,393 pieces of data were used for model testing. The remaining 26,706 pieces of data were used for model validation. The ratio of training set, test set, and validation set are shown in Figure 4.

In this paper, the principal component analysis method is used to extract the common factors that characterize the hypnotic state by the road to the greatest extent. Based on sorting and analysis of the preprocessed experimental data, a principal component analysis model is constructed to achieve the purpose of dimensionality reduction and avoid the problem of dimensionality.

The principal component analysis of the vehicle driving experimental data is as follows.

The KMO and Bartlett test on the data of the vehicle driving experiment were first carried out to test whether it was suitable for the principal component analysis method, as shown in Table 3. According to the test results, the KMO coefficient is 0.835, and the significance is 0.000. Therefore, the principal component analysis method is suitable for the vehicle driving experimental dataset.

**Figure 5:**
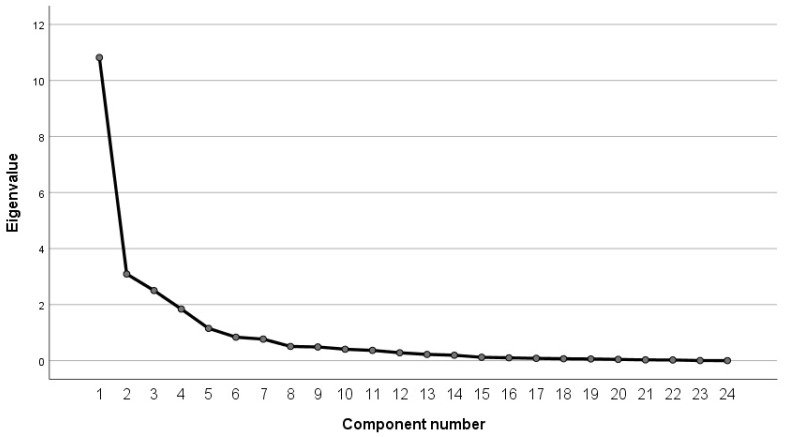
Lithograph of the vehicle driving experimental data.

The results shown in Table 4 and Table 5 and Figure 5 intuitively present the loading square, variance percentage, and the information content of each factor. The analysis results show that the eigenvalues of the first five components are all greater than 1, and the cumulative variance contribution rate reached 80.846%, higher than 80%, which can represent most of the information on all parameters. Considering the information content of each component and the inflection point of the gravel map, in this paper, the first five components (respectively, denoted as C1, C2, C3, C4, and C5) are selected as the main features of road hypnotic state identification in the vehicle driving experimental dataset. The formula for calculating its coefficient is $U_{i}=\frac{A_{i}}{\sqrt{\lambda_{i}}}$. $U_{i}$ is the factor loading coefficient matrix of each component. $A_{i}$ is the factor loading value corresponding to each component in the component matrix. $\lambda_{i}$ is the eigenvalue corresponding to each component in the total variance explanation table. The final calculation formula for each component is as follows:

(5) $$\begin{array}{l} C1=0.062GV+0.038P1+0.051P2+0.04P3+0.05P4\\ +\;0.024I+0.077G1+0.077G2+0.079G3+0.078G4\\ +\;0.073G5+0.069G6+0.04G7+0.07G8+0.073G9\\ +\;0.053G10+0.067G11+0.073G12+0.012F1\\ +\;0.034F2+0.07P5+0.07P6+0.07P7+0.07P8\\ C2=0.013GV+0.181P1+0.19P2-0.102P3-0.12P4\\ -\;0.024I+0.043G1-0.046G2+0.055G3+0.29G4\\ -\;0.097G6+0.14G7+0.063G8+0.151G9-0.125G10\\ -\;0.12G11-0.15G12+0.047F1-0.092F2+0.166P5\\ +\;0.158P6-0.155P7-0.159P8\\ C3=-0.029GV+0.173P1+0.1P2+0.282P3+0.241P4\\ +\;0.183I+0.023G1-0.162G2-0.02G3-0.114G4\\ +\;0.077G5-0.166G6+0.005G7-0.14G8-0.049G9\\ -\;0.008G10-0.117G11+0.054G12+0.006F1-0.243F2\\ +\;0.024P5+0.012P6+0.098P7+0.081P8\\ C4=0.021GV-0.113P1-0.102P2+0.022P3+0.012P4\\ +\;0.017I+0.208G1+0.005G2+0.189G3+0.055G4\\ +\;0.162G5-0.044G6-0.338G7+0.018G8-0.031G9\\ -\;0.28G10-0.055G11-0.038G12+0.455F1-0.013F2\\ -\;0.02P5-0.034P6-0.02P7-0.033P8\\ C5=-0.162GV+0.323P1+0.061P2-0.185P3-0.183P4\\ +\;0.531I-0.105G1+0.072G2-0.104G3-0.113G4\\ -\;0.083G5+0.192G6-0.235G7-0.195G8-0.19G9\\ -\;0.084G10+0.174G11-0.004G12+0.07F1+0.25F2\\ +\;0.183P5+0.173P6+0.043P7+0.052P8 \end{array}$$

The ratio of the eigenvalues corresponding to the obtained five principal components to the total eigenvalues of the extracted principal components is used as the weight to calculate the principal component comprehensive model. The calculation formula is as follows:

(6) $$C=\sum_{n=1}^{5}\frac{\lambda_{n}}{\sum_{i=1}^{5}\lambda_{i}}C_{n}$$

The principal component analysis of the simulated driving test data is as follows.

For the data of the simulated driving experiment, the KMO and Bartlett test were also adopted to test whether it was suitable for the principal component analysis method, as shown in Table 6. According to the test results, the KMO coefficient is 0.847, and the significance is 0.000. Therefore, the principal component analysis method is suitable for the vehicle driving experimental dataset.

**Figure 6:**
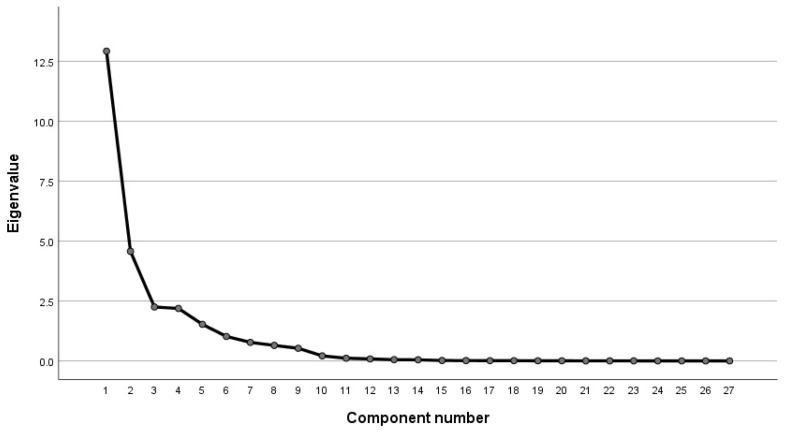
Lithograph of the simulated driving test data.

The results shown in Table 7 and Table 8 and Figure 6 intuitively present the loading square, variance percentage, and the information content of each factor. The analysis results show that the eigenvalues of the first five components are all greater than 1, and the cumulative variance contribution rate reached 90.652%, higher than 90%, which can represent most of the information of all parameters. Considering the information content of each component and the inflection point of the gravel map, in this paper, the first six components are selected as the main features of road hypnotic state identification in the vehicle driving experimental dataset. The formula for calculating its coefficient is $U_{i}=\frac{A_{i}}{\sqrt{\lambda_{i}}}$. $U_{i}$ is the factor loading coefficient matrix of each component. $A_{i}$ is the factor loading value corresponding to each component in the component matrix. $\lambda_{i}$ is the eigenvalue corresponding to each component in the total variance explanation table. The final calculation formula for each component is as follows:

(7) $$\begin{array}{l} C1=-0.003GV+0.06P1+0.049P2+0.057P3+0.048P4\\ -\;0.048I+0.069G1+0.07G2+0.069G3+0.069G4\\ +\;0.07G5+0.071G6-0.049G7+0.058G8-0.045G10\\ +\;0.061G11+0.017G12+0.069F1+0.07F2\\ +\;0.068P5+0.039P6+0.069P7+0.045P8\\ C2=0.025GV+0.057P1+0.077P2+0.052P3+0.064P4\\ +\;0.163I-0.091G1+0.073G2-0.094G3+0.077G4-0.088G5\\ -\;0.068G6+0.067G7+0.018G8-0.124G9-0.012G10\\ +\;0.063G11-0.152G12-0.091F1+0.07F2-0.098P5\\ +\;0.163P6-0.093P7+0.154P8\\ C3=-0.033GV+0.24P1+0.274P2+0.269P3+0.295P4\\ -\;0.014I-0.03G1-0.059G2-0.028G3-0.068G4\\ -\;0.031G5-0.051G6+0.168G7+0.006G8+0.123G9\\ +\;0.205G10-0.058G11+0.072G12-0.029F1-0.062F2\\ -\;0.03P5-0.121P6-0.028P7-0.113P8\\ C4=0.027GV+0.064P1+0.078P2+0.059P3+0.087P4\\ -\;0.061I+0.007G1-0.045G2+0.003G3-0.047G4\\ +\;0.011G5-0.043G6-0.061G7-0.164G8-0.113G9\\ -\;0.143G10-0.049G11-0.097G12+0.007F1-0.046F2\\ +\;0.024P5-0.081P6-0.01P7-0.086P8\\ C5=-0.194GV-0.066P1-0.084P2-0.054P3-0.087P4\\ +\;0.096I-0.048G1-0.067G2-0.045G3+0.073G4\\ -\;0.051G5+0.061G6+0.129G7+0.254G8+0.217G9\\ +\;0.257G10+0.11G11+0.228G12-0.046F1+0.073F2\\ -\;0.063P5+0.097P6-0.023P7+0.071P8\\ C6=0.824GV-0.028P1-0.059P2-0.022P3-0.044P4\\ +\;0.012I+0.028G1+0.07G2+0.026G3+0.072G4\\ +\;0.024G5+0.066G6+0.167G7+0.002G8+0.293G9\\ +\;0.224G10-0.09G11+0.128G12+0.028F1+0.069F2\\ +\;0.012P5+0.048P6+0.029P7+0.04P8 \end{array}$$

The ratio of the eigenvalues corresponding to the obtained six principal components to the sum of the eigenvalues of the extracted principal components is used as the weight to calculate the principal component comprehensive model. The calculation formula is as follows:

(8) $$C=\sum_{n=1}^{6}\frac{\lambda_{n}}{\sum_{i=1}^{6}\lambda_{i}}C_{n}$$

### 4.2. Model Training, Testing, Validation, and Evaluation Results

**Figure 7:**
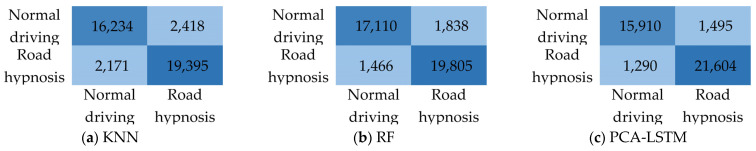
Confusion matrix of vehicle driving experiments.

**Figure 8:**
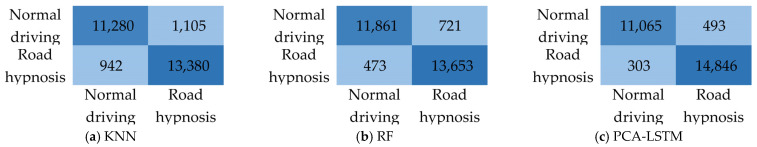
Confusion matrix of simulated driving experiments.

The data and labels collected from simulation experiments and vehicle driving experiments are used for model calibration, training, and verification. PyCharm2021 is adopted for algorithm programming. The training set is used to train the PCA-LSTM identification model. The PCA algorithm is used to extract the principal component features, which are substituted into the LSTM neural network identification model to establish the PCA-LSTM road hypnosis identification model. Considering the objective and multi-dimensional measurement model indicators, in this paper, simulated driving data and vehicle driving data are adopted to verify the trained PCA-LSTM model. To verify the superiority of the optimized recognition algorithm, the random forest and K-nearest neighbor algorithms are introduced to compare with the PCA-LSTM model. The base evaluator of the random forest is a decision tree model. The core idea of the bagging method is to construct multiple independent base evaluators, and determine the classification result of the final integrated evaluator through the principle of voting or majority voting. This model reduces the problem of easily overfitting with the decision tree model to a certain extent. At the same time, it is simple in principle and easy to operate, so it is widely used in data analysis, processing, and other fields. The K-nearest neighbor algorithm (KNN) is a supervised learning model. When the input new data are predicted, the KNN algorithm predicts them by majority voting and judges that they belong to the category with the greatest frequency of appearance in the K categories closest to the new data. The two most important factors of the KNN algorithm are the selection of the K value and the calculation of the distance. The KNN classification algorithm has the advantages of simple principles and good classification effects and is widely used in feature classification tasks. The confusion matrix of the obtained results is shown in Figure 7 and Figure 8 and Table 9.

To evaluate the performance and generalization ability of the road hypnosis identification model, the performance of the PCA-LSTM, random forest, and K-nearest neighbor models is evaluated using four evaluation indicators: accuracy, recall, precision, and F1-score. Accuracy is the percentage of the number of correctly identified road hypnotic states and non-road hypnotic states to the total number of behaviors in the experimental data—that is, the probability of correct identification. Its calculation formula is as follows:

(9) $$Accuracy=\frac{TP+TN}{FP+FN+TP+TN}\times 100\%$$

Among them, TP is the number of true positive samples. TN is the number of true negative samples. FP is the number of false positive samples. FN is the number of false negative samples.

Recall is the ratio of the number of correctly classified positive samples to the total number of positive samples. There is a trade-off between recall and precision, which represents the balance between the need to capture the minority class and the need to avoid misjudging the majority class. Its calculation formula is as follows:

(10) $$Recall=\frac{TP}{TP+FN}\times 100\%$$

Precision is the ratio of the number of correctly classified positive samples to the total number of normal driving samples predicted by the model. The accuracy rate represents the measure of the cost required to judge the majority class wrong.

(11) $$Precison=\frac{TP}{TP+FP}\times 100\%$$

Due to mutual constraints between precision and recall, in this study, the comprehensive indicator of the F1-score is introduced to reconcile the balance between them. The value range of the F1-score is [0, 1], and the higher the score, the better the performance.

(12) $$F1-score=\frac{2\times Pre\times Rec}{Pre+Rec}\times 100\%$$

**Figure 9:**
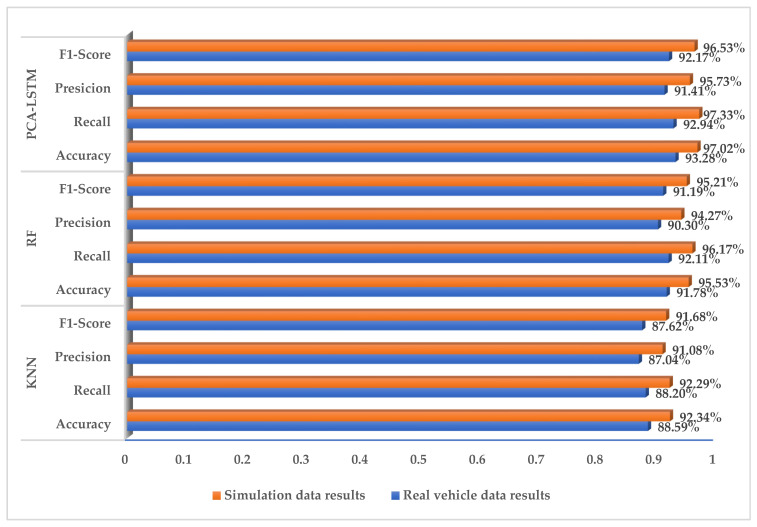
Vehicle data results and simulation data results.

The verification results of the three types of identification models are calculated according to the confusion matrix, as shown in Figure 9.

## 5. Discussion

It can be seen from the confusion matrix in Table 9 that normal driving misclassified as road hypnosis has a larger number than that of road hypnosis misjudged as normal driving. The reason for this result may be related to the calibration of the dataset, because road hypnosis with a lesser degree or samples before and after the appearance of road hypnosis will be marked as road hypnosis during manual calibration. The number of road hypnosis states identified by the PCA-LSTM model is the largest, which also shows the superiority of the PCA-LSTM model in road hypnosis identification. However, in the results of KNN and RF model identification, there are more misjudgments in both normal driving and road hypnosis than that of the PCA-LSTM model, and its performance in road hypnosis detection in this study is average.

It can be seen from Figure 9 that the KNN model has the lowest accuracy rate of road hypnosis identification and the worst generalization ability, and is the worst-performing model among the three types of models. Although the recall rate and F1-score value of the RF model are slightly different from those of the PCA-LSTM model, there is a significant gap in the accuracy rate, and the overall performance of the model is unstable. Compared with the KNN and RF models, the four evaluation indicators of the PCA-LSTM model proposed in this paper have superior performance, and they can accurately identify road hypnosis in simulated driving experiments and vehicle driving experiments. This is because tunnels and highways, as typical monotonous scenes, are very likely to induce road hypnosis. Therefore, the characteristic parameters of the road hypnosis collected from the vehicle driving experiment are more typical. Based on the data from vehicle driving experiments, an accurate and effective road hypnosis identification model can be established. In the simulated driving experiment, we set up a more typical monotonous scene. Compared with the vehicle driving experiment, there are fewer interference factors, which are easier to be controlled during the experiment, and it can more accurately induce the driver’s road hypnosis and collect corresponding parameters. Therefore, the performance of various models established by the data obtained from the simulated driving test is significantly better than that of the vehicle driving test.

Since the external characteristics of cognitive distraction and road hypnosis are similar, there may be cases where the state of cognitive distraction is misjudged as the state of road hypnosis. However, the internal mechanism of cognitive distraction and road hypnosis is different. Cognitive distraction means that the driver’s cognitive resources and attention are occupied by the driving subtask instead of focusing on the main driving task. Although the driver did not focus on the main task of driving under road hypnosis, the attention of cognitive resources was not occupied by other certain secondary tasks. The experimental methods employed in this study differ from those commonly used in studies of cognitive distraction. During the driving process, we did not set secondary tasks for the driver to induce road hypnosis but placed the driver in a natural driving state. By observing the changes in ECG and eye movements, it is possible to detect the road hypnosis that the driver may experience. However, in cognitive distraction research, cognitive distraction secondary tasks (such as working memory tasks, etc.) are often used, and the experimenter actively asks the driver to perform secondary tasks to induce cognitive distraction during the driving process. Therefore, in the experimental method of this study, the interference of cognitive distraction on the experimental results was excluded as much as possible.

ECG and eye movement features were selected in this study. Whether road hypnosis has occurred can be determined by the driver’s external performance and active inquiry. When road hypnosis appears, although the driver usually cannot perceive subjectively that he or she is in road hypnosis, it is a real existing unconscious driving state. However, when the driver emerges from road hypnosis, it is often accompanied by a marked state of alertness. The time that we chose to question them was during this alert state, which not only achieved the purpose of accurately recording the hypnotic state by the road but also avoided the interference caused by active inquiry into the driving process. Drivers may not remember the experience during their road hypnosis, but they often recognise that they have recently experienced road hypnosis. We inquired in real time when suspected road hypnosis during driving was observed, to avoid the situation of complete forgetfulness or inaccurate memory when inquiring about the driver only through post-event video. The results prove that our experimental method is feasible and the experimental procedure is reasonable.

There are also some shortcomings in this study. (1) Road hypnosis can easily appear repeatedly during driving. For some milder or shorter-duration road hypnotic states, the driver’s external performance is insufficient. This lesser and recurring condition is difficult to be detected by electrocardiogram and eye movement alone. (2) Although we attempted to eliminate the interference caused by cognitive distraction from the experimental method, it is rather impossible to directly distinguish road hypnosis from cognitive distraction according to the characteristics of eye movement. It is difficult to completely rule out the influence of cognitive distraction on the experimental results only by using eye movement features. (3) During the experiment, no additional requirements were placed on the drivers. This was in order to place the driver in a natural driving state. However, our road hypnosis identification model is only established under experimental conditions. Although the combination of a vehicle driving experiment and simulation experiment is adopted, corresponding identification models are established according to the data obtained from the vehicle driving experiment and simulation experiment, respectively, and no unified model is formed. (4) Two typical monotonous scenes of the tunnel and highway were selected for the experiment, but road hypnosis does not only occur in monotonous scenes. Regardless of whether the scene is familiar (especially the repeated scene experienced in daily life), or non-familiar to the driver, if the same or a similar scene continues to appear in a certain section of the road, road hypnosis is also extremely likely to appear. (5) In vehicle driving experiments, there is currently a lack of vehicle driving experimental verification of the expressway scene. This is because the speed of vehicles driving on the expressway is relatively fast, and it is difficult to guarantee the safety of the vehicle driving experiment. (6) When determining whether the driver is in a state of distraction, in this study, the changes in ECG law combined with the real-time observation and questioning of experimental assistants were used, which interfered with the driver’s driving process to a certain extent.

This study was an exploratory study on road hypnotic state identification. In further research, other sensing methods (such as near-brain infrared and EEG equipment) can be considered to study the law of brain activity of drivers under road hypnosis and to deeply analyze its internal mechanism. In terms of experimental methods, the external representation and internal mechanism of the typical road hypnosis can be adopted for the extraction of characteristic parameters during the driving experiment, to avoid the interference caused by the interrogation of the experimental assistants in the driving process. This can be verified by vehicle driving experiments on closed expressway sections, and further experiments can also be carried out on general road scenes, especially those familiar to drivers. Considering the simulated driving test and the vehicle driving test comprehensively, a unified road hypnosis identification model is expected to be established.

## 6. Conclusions

In this paper, the driver’s normal driving state and road hypnosis are classified by collecting and classifying the driver’s eye movement information in two typical monotonous scenes of a tunnel and highway. A road hypnosis identification model based on PCA-LSTM is proposed. The main work of this paper includes the following aspects:

(1) Simulated driving experiments and vehicle driving experiments were designed and organized. A total of 50 test subjects were recruited to collect experimental data on drivers’ normal driving and road hypnosis. After the experiment, the video during the experiment was judged through active inquiry and the expert scoring method during the experiment. The tag data of drivers in different states were recorded, and a road hypnosis sample database was established, preparing for the analysis of road hypnosis characteristic parameters and the construction and calibration of road hypnosis identification models. A variety of eye movement characteristic parameters contained in the road hypnosis sample database help to make up for the poor stability and robustness of the identification model caused by a single eye movement characteristic parameter.
(2) The method of adopting principal component analysis to extract the principal components of road hypnosis characteristic parameters was introduced. A principal component analysis model was constructed, in which the principal components that met the conditions were extracted as the characteristic parameters, to reduce the data dimension. Through the principal component analysis model, the comprehensive principal components of the vehicle driving test and those of the simulated driving test were extracted, which were adopted as the input parameters for the subsequent establishment of the LSTM model.
(3) A road hypnosis identification model was established by using the LSTM algorithm. To verify the validity and accuracy of the model, it was compared with the model established by the KNN and RF algorithms. Four evaluation indicators of accuracy, recall, precision, and F1-score were introduced for model evaluation. The verification results show that the PCA-LSTM model established in this paper has good generalization ability and stability, and it can accurately identify road hypnosis in simulated driving experiments and vehicle driving experiments. This study demonstrates the existence of road hypnosis and the feasibility of detecting road hypnosis by machine learning methods. It provides a reliable method and technical support for the real-time and accurate identification of road hypnosis, which is of great significance for improving the active safety performance of smart cars.

## Figures and Tables

**Table 1:** Basic information about drivers.

	Range (Year)	Number of Drivers
Age	30–40	26
	41–50	18
	51–60	6
Driving age	10–15	11
	16–20	18
	21–32	21

**Table 2:** Eye tracker acquisition parameters and their meanings.

Type	Name	Explanation
Basic Information	Recording Timestamp (ms)	Timestamp of eye tracking data
	Time of Day (HH: mm: SS: ms)	Recording time of eye movement data
	Stimulus	Recording name
	Participant	Participant name
	Tracker Ratio (%)	The ratio of the captured effective gaze point to the frame rate
	Video Time (HH: mm: SS: ms)	Recording time relative to video recording start time
Pupil Information	Validity Left	Validity of left eye pupil recognition: 0 means the recognition is successful; 1 means the recognition fails
	Validity Right	Validity of right eye pupil recognition: 0 means the recognition is successful; 1 means the recognition fails
	Pupil Diameter Left (mm)	Left eye pupil diameter
	Pupil Diameter Right (mm)	Right eye pupil diameter
	IPD (mm)	Interpupillary distance
Gaze Point Information	Gaze Point Index	Original gaze point index
	Gaze Point X (px)	Original fixation x-coordinate in pixels
	Gaze Point Y (px)	Original fixation y-coordinate in pixels
	Gaze Point Right X (px)	X-coordinate of the original gaze point of the right eye
	Gaze Point Right Y (px)	Y-coordinate of the original gaze point of the right eye
	Gaze Point Left X (px)	X-coordinate of the original gaze point of the left eye
	Gaze Point Left Y (px)	Y-coordinate of the original gaze point of the left eye
	Fixation Index	Aggregate gaze index
	Fixation Point X (px)	The x-coordinate of the gaze point in pixels
	Fixation Point Y (px)	The y-coordinate of the gaze point in pixels
	Fixation Duration (ms)	Gaze duration
	Gaze Vector Left X	The x-coordinate of the left eye gaze vector
	Gaze Vector Left Y	The y-coordinate of the left eye gaze vector
	Gaze Vector Left Z	The z-coordinate of the left eye gaze vector
	Gaze Vector Right X	The x-coordinate of the right eye gaze vector
	Gaze Vector Right Y	The y-coordinate of the right eye gaze vector
	Gaze Vector Right Z	The z-coordinate of the left eye gaze vector
Saccade Information	Saccade Index	Saccade index
	Saccade Amplitude (px)	Saccade amplitude, angle value
	Saccade Velocity Average (px/ms)	Average velocity of saccade
	Saccade Single Velocity (px/ms)	Average saccade velocity per frame
	Saccade Velocity Peak (px/ms)	Velocity peaks during saccades
Blink Information	Blink Index	Blink index
	Blink Duration (ms)	Duration of blink
	Blink Eye	Blink label: 0 for double wink; 1 for left wing; 2 for right wing

**Table 3:** KMO and Bartlett test of the vehicle driving experimental data.

**KMO Sampling Suitability Quantity**	0.835	
**Bartlett’s sphericity test**	Approximate chi-square	7,735,048.806
	Degrees of freedom	276
	Salience	0.000

**Table 4:** Total explained variance of the vehicle driving experimental data.

	Initial Eigenvalues			Extracted Load Sum of Squares		
Component	Total	Percent Variance (%)	Accumulation (%)	Total	Percent Variance (%)	Accumulation (%)
1	10.818	45.075	45.075	10.818	45.075	45.075
2	3.092	12.884	57.959	3.092	12.884	57.959
3	2.502	10.425	68.385	2.502	10.425	68.385
4	1.841	7.669	76.054	1.841	7.669	76.054
5	1.150	4.792	80.846	1.150	4.792	80.846
6	0.838	3.490	84.336			
7	0.768	3.198	87.534			
8	0.507	2.112	89.646			
9	0.487	2.031	91.677			
10	0.407	1.694	93.371			
11	0.366	1.527	94.898			
12	0.282	1.173	96.071			
13	0.222	0.924	96.996			
14	0.193	0.806	97.801			
15	0.119	0.498	98.299			
16	0.100	0.417	98.716			
17	0.082	0.341	99.057			
18	0.068	0.285	99.342			
19	0.059	0.247	99.590			
20	0.044	0.182	99.772			
21	0.027	0.113	99.884			
22	0.023	0.097	99.982			
23	0.003	0.011	99.993			
24	0.002	0.007	100.000			

**Table 5:** Composition matrix of the vehicle driving experimental data.

	Component					
	Representation Symbol	1	2	3	4	5
Gaze Velocity	GV	0.673	0.040	−0.072	0.038	−0.186
Pipul Diameter Left [px]	P1	0.412	0.561	0.432	−0.208	0.371
Pipul Diameter Left [mm]	P2	0.555	0.587	0.250	−0.188	0.070
Pipul Diameter Right [px]	P3	0.432	−0.314	0.704	0.041	−0.213
Pipul Diameter Right [mm]	P4	0.545	−0.372	0.604	0.022	−0.210
IPD [mm]	I	0.258	−0.075	0.457	0.032	0.611
Gaze Point X [px]	G1	0.837	0.132	0.058	0.383	−0.120
Gaze Point Y [px]	G2	0.838	−0.144	−0.405	0.009	0.083
Gaze Point Left X [px]	G3	0.854	0.171	−0.050	0.347	−0.120
Gaze Point Left Y [px]	G4	0.848	0.091	−0.285	0.102	−0.130
Gaze Point Right X [px]	G5	0.790	0.001	0.192	0.298	−0.096
Gaze Point Right Y [px]	G6	0.746	−0.300	−0.417	−0.081	0.220
Gaze Vector Left X	G7	0.428	0.433	0.013	−0.622	−0.270
Gaze Vector Left Y	G8	0.755	0.196	−0.350	0.032	−0.225
Gaze Vector Left Z	G9	0.791	0.468	−0.122	−0.056	−0.219
Gaze Vector Right X	G10	0.570	−0.387	−0.021	−0.514	−0.097
Gaze Vector Right Y	G11	0.724	−0.371	−0.292	−0.101	0.200
Gaze Vector Right Z	G12	0.787	−0.463	0.135	−0.070	−0.004
Fixation Point X [px]	F1	0.128	0.145	0.014	0.838	0.081
Fixation Point Y [px]	F2	0.371	−0.284	−0.608	−0.024	0.287
Pupil Position Left X	P5	0.754	0.514	0.061	−0.037	0.210
Pupil Position Left Y	P6	0.760	0.490	0.031	−0.063	0.199
Pupil Position Right X	P7	0.760	−0.480	0.244	−0.037	0.049
Pupil Position Right Y	P8	0.762	−0.491	0.203	−0.061	0.059

**Table 6:** KMO and Bartlett test of the simulated driving test data.

**KMO Sampling Suitability Quantity**	0.847	
**Bartlett’s sphericity test**	Approximate chi-square	840,279.813
	Degrees of freedom	351
	Salience	0.000

**Table 7:** Total explained variance of the simulated driving test data.

	Initial Eigenvalues			Extracted Load Sum of Squares		
Component	Total	Percent Variance (%)	Accumulation (%)	Total	Percent Variance (%)	Accumulation (%)
1	12.920	47.851	47.851	12.920	47.851	47.851
2	4.571	16.929	64.780	4.571	16.929	64.780
3	2.249	8.329	73.110	2.249	8.329	73.110
4	2.189	8.107	81.217	2.189	8.107	81.217
5	1.526	5.652	86.869	1.526	5.652	86.869
6	1.021	3.783	90.652	1.021	3.783	90.652
7	0.772	2.860	93.512			
8	0.647	2.397	95.909			
9	0.528	1.954	97.863			
10	0.211	0.782	98.644			
11	0.112	0.414	99.058			
12	0.084	0.313	99.371			
13	0.052	0.193	99.564			
14	0.047	0.173	99.738			
15	0.018	0.067	99.805			
16	0.012	0.046	99.851			
17	0.012	0.043	99.893			
18	0.009	0.034	99.927			
19	0.006	0.021	99.948			
20	0.005	0.017	99.965			
21	0.003	0.010	99.976			
22	0.003	0.010	99.985			
23	0.002	0.008	99.993			
24	0.002	0.006	99.999			
25	0.000	0.001	100.000			
26	8.708 × 10^−5^	0.000	100.000			
27	4.389 × 10^−5^	0.000	100.000			

**Table 8:** Composition matrix of the simulated driving test data.

	Component					
	Representation Symbol	1	2	3	4	5
Gaze Velocity	GV	−0.038	0.113	−0.074	0.058	−0.295
Pipul Diameter Left [px]	P1	0.771	0.263	0.540	0.141	−0.101
Pipul Diameter Left [mm]	P2	0.639	0.351	0.616	0.170	−0.128
Pipul Diameter Right [px]	P3	0.734	0.237	0.606	0.129	−0.082
Pipul Diameter Right [mm]	P4	0.625	0.293	0.664	0.191	−0.132
IPD [mm]	I	−0.624	0.743	−0.031	−0.133	0.146
Gaze Point X [px]	G1	0.067	0.039	−0.140	0.867	0.462
Gaze Point Y [px]	G2	0.894	−0.417	−0.066	0.016	−0.073
Gaze Point Left X [px]	G3	0.909	0.334	−0.134	−0.099	0.102
Gaze Point Left Y [px]	G4	0.886	−0.431	−0.064	0.007	−0.068
Gaze Point Right X [px]	G5	0.892	0.352	−0.153	−0.102	0.111
Gaze Point Right Y [px]	G6	0.899	−0.404	−0.069	0.025	−0.078
Gaze Vector Left X	G7	0.915	0.311	−0.114	−0.095	0.093
Gaze Vector Left Y	G8	−0.633	0.307	0.378	−0.133	0.197
Gaze Vector Left Z	G9	0.744	0.083	0.014	−0.359	0.387
Gaze Vector Right X	G10	−0.006	−0.566	0.276	−0.246	0.331
Gaze Vector Right Y	G11	−0.575	−0.057	0.462	−0.313	0.393
Gaze Vector Right Z	G12	0.792	0.290	−0.129	−0.108	0.167
Fixation Point X [px]	F1	0.221	−0.693	0.163	−0.213	0.349
Fixation Point Y [px]	F2	0.065	0.034	−0.098	0.887	0.432
Pupil Position Left X	P5	0.069	−0.337	0.137	−0.232	0.506
Pupil Position Left Y	P6	0.893	−0.415	−0.065	0.015	−0.070
Pupil Position Right X	P7	0.907	0.321	−0.138	−0.101	0.111
Pupil Position Right Y	P8	0.874	−0.448	−0.067	0.052	−0.095

**Table 9:** Validation results of the model.

**Model Predictions**	**Real Data Results**					
	**KNN**		**RF**		**PCA-LSTM**	
	**Normal Driving**	**Road Hypnosis**	**Normal Driving**	**Road Hypnosis**	**Normal Driving**	**Road Hypnosis**
**Normal Driving**	16,234	2418	17,110	1838	15,910	1495
**Road Hypnosis**	2171	19,395	1466	19,805	1209	21,604
**Model Predictions**	**Simulation Data Results**					
	**KNN**		**RF**		**PCA-LSTM**	
	**Normal Driving**	**Road Hypnosis**	**Normal Driving**	**Road Hypnosis**	**Normal Driving**	**Road Hypnosis**
**Normal Driving**	11,280	1105	11,861	721	11,065	493
**Road Hypnosis**	942	13,380	473	13,653	303	14,846

## Data Availability

The data presented in this study are available on request from the corresponding author. The data are not publicly available due to privacy.

## References

[1] Adanu E.K., Smith R., Powell L., Jones S. (2017). Multilevel analysis of the role of human factors in regional disparities in crash outcomes. Accid. Anal. Prev..

[2] Haghighattalab S., Chen A., Fan Y., Mohammadi R. (2019). Engineering ethics within accident analysis models. Accid. Anal. Prev..

[3] Wang X.Y., Liu Y.Q., Guo Y.Q., Xia Y.Y., Wu C.Z. (2019). Transformation mechanism of vehicle cluster situations under dynamic evolution of driver’s propensity. Transp. Res. F Traffic Psychol. Behav..

[4] Wang X.Y., Liu Y.Q., Wang J.Q., Zhang J.L. (2019). Study on influencing factors selection of driver’s propensity. Transp. Res. D Transp. Environ..

[5] Wang X.Y., Liu Y.Q., Wang F., Wang J., Liu L., Wang J. (2019). Feature extraction and dynamic identification of drivers’ emotions. Transp. Res. F Traffic Psychol. Behav..

[6] Liu Y.Q., Wang X.Y. (2020). The analysis of the driver’s behavioral tendency under different emotional stated based on a Bayesian Network. IEEE Trans. Affect Comput..

[7] Wang X.Y., Guo Y.Q., Bai C.L., Yuan Q., Liu S.L., Han J.Y. (2020). Driver’s intention identification with involvement of emotional factors in two-lane roads. IEEE Trans. Intell. Transp..

[8] Tosi J.D., Ledesma R.D., Lázaro C.M.D., Poo F.M. (2020). Implicit attitudes towards risky driving behaviors: Evidence of validity for the implicit association test. J. Saf. Res..

[9] Yang S., Wang W., Jiang Y., Wu J., Zhang S., Deng W. (2019). What contributes to driving behavior prediction at unsignalized intersections?. Transp. Res. C Emerg. Technol..

[10] Yan Y., Yuan H.Z., Yang X.L., Liu G., Guo Z.Y., Wang L. (2021). A model of the relationship between monotonic road environment and driving fatigue based on multi-source data. China J. Highw. Transp..

[11] Salvati L., d’Amore M., Fiorentino A., Pellegrino A., Sena P., Villecco F. (2021). On-road detection of driver fatigue and drowsiness during medium-distance journeys. Entropy.

[12] Kaber D., Jin S., Zahabi M., Pankok C. (2016). The effect of driver cognitive abilities and distractions on situation awareness and performance under hazard condition. Transp. Res. F Traffic Psychol. Behav..

[13] Alexey K., Roman S., Christian K., Alexander S. (2021). Driver distraction detection methods: A literature review and framework. IEEE Access.

[14] Femke C., Pieter V.D., Jan D.H. (2022). Predicting drunk driving, using a variant of the implicit association test. J. Saf. Res..

[15] Kadoya Y., Watanapongvanich S., Khan M. (2021). How is an emotion associated with driving speed? A study of taxi drivers in Japan. Transp. Res. F Traffic Psychol. Behav..

[16] Hasan B.M.S., Abdulazeez A.M. (2021). A review of principal component analysis algorithm for dimensionality reduction. J. Soft Comput. Data Min..

[17] Hochreiter S., Schmidhuber J. (1997). Long short-term memory. Neural Comput..

[18] Griffith W.W. (1963). Highway hypnosis: An hypothesis. Int. J. Clin. Exp. Hypn..

[19] Shor R.E., Thackray R.I. (1970). A program of research in “highway hypnosis”: A preliminary report. Accid. Anal. Prev..

[20] Griffith W.W., Shor R.E. (1970). An historical note on highway hypnosis. Accid. Anal. Prev..

[21] Wertheim A.H. (1978). Explaining highway hypnosis: Experimental evidence for the role of eye movements. Accid. Anal. Prev..

[22] Cerezuela G.P., Tejero P., Choliz M., Chisvert M., Monteagudo M.J. (2004). Wertheim’s hypothesis on ‘highway hypnosis’: Empirical evidence from a study on motorway and conventional road driving. Accid. Anal. Prev..

[23] Gers F.A., Schmidhuber J., Cummins F.A. (2000). Learning to Forget: Continual Prediction with LSTM. Neural Comput..

[24] Gers F.A., Schmidhuber J. Recurrent nets that time and count. Proceedings of the IEEE-INNS-ENNS International Joint Conference on Neural Networks.

[25] Graves A., Schmidhuber J. (2005). Framewise phoneme classification with bidirectional LSTM and other neural network architectures. Neural Netw..

[26] Mendi A.F. (2022). A sentiment analysis method based on a blockchain-supported long short-term memory deep network. Sensors.

[27] Alameen S.A., Alhothali A.M. (2023). A lightweight driver drowsiness detection system using 3DCNN with LSTM. Comput. Syst. Sci. Eng..

[28] Cura A., Kucuk H., Ergen E., Oksuzoglu I.B. (2021). Driver profiling using long short term memory (LSTM) and convolutional neural network (CNN) methods. IEEE Trans. Int. Transp. Syst..

[29] Wang S., Zhao X., Yu Q., Yuan T. (2020). Identification of driver braking intention based on long short-term memory (LSTM) network. IEEE Access.

[30] Wollmer M., Blaschke C., Schindl T., Schuller B., Farber B., Mayer S., Trefflich B. (2011). Online driver distraction detection using long short-term memory. IEEE Trans. Int. Transp. Syst..

[31] Wu Z.Q., Liang K.C., Liu D.C., Zhao Z.G. (2022). Driver lane change intention recognition based on Attention Enhanced Residual-MBi-LSTM network. IEEE Access.

